# Evaluation of a new suture material (Duramesh™) by measuring suture tension in small and large bites techniques for laparotomy closure in a porcine model

**DOI:** 10.1007/s10029-020-02140-7

**Published:** 2020-02-21

**Authors:** Y. Yurtkap, F. P. J. den Hartog, W. van Weteringen, J. Jeekel, G. J. Kleinrensink, J. F. Lange

**Affiliations:** 1 Department of Surgery, Erasmus University Medical Centre, Dr. Molewaterplein 40, 3015 GD Rotterdam, The Netherlands; 2 Department of Paediatric Surgery, Sophia Children’s Hospital, Erasmus University Medical Centre, Rotterdam, The Netherlands; 3 Department of Neuroscience and Anatomy, Erasmus University Medical Centre, Rotterdam, The Netherlands

**Keywords:** Suture tension, Biomedical sensor, Incisional hernia, Laparotomy closure

## Abstract

**Purpose:**

After closure of laparotomies, sutures may pull through tissue due to too high intra-abdominal pressure or suture tension, resulting in burst abdomen and incisional hernia. The objective of this study was to measure the suture tension in small and large bites with a new suture material.

**Methods:**

Closure of the *linea alba* was performed with small bites (i.e., 5 mm between two consecutive stitches and 5 mm distance from the incision) and large bites (i.e., 10 mm × 10 mm) with Duramesh™ size 0 (2 mm) and PDS II 2-0 in 24 experiments on six porcine abdominal walls. The abdominal wall was fixated on an artificial computer-controlled insufflatable abdomen, known as the ‘AbdoMan’. A custom-made suture tension sensor was placed in the middle of the incision.

**Results:**

The suture tension was significantly lower with the small bites technique and Duramesh™ when compared with large bites (small bites 0.12 N (IQR 0.07–0.19) vs. large bites 0.57 N (IQR 0.23–0.92), *p*  < 0.025). This significant difference was also found in favour of the small bites with PDS II 2-0 (*p*  < 0.038). No macroscopic tissue failure was seen during or after the experiments.

**Conclusion:**

Closure of the abdominal wall with the small bites technique and Duramesh^™^ was more efficient in dividing suture tension across the incision when compared to large bites. However, suture tension compared to a conventional suture material was not significantly different, contradicting an advantage of the new suture material in the prevention of burst abdomen and incisional hernia during the acute, postoperative phase.

**Electronic supplementary material:**

The online version of this article (10.1007/s10029-020-02140-7) contains supplementary material, which is available to authorized users.

## Introduction

Abdominal wound dehiscence (burst abdomen, ‘Platzbauch’) has an incidence of up to 4% and it is a feared early complication after abdominal surgery with sequelae like evisceration, prolonged hospitalization and high mortality rates [1]. In addition, incisional hernia is a common complication after midline incisions with a 5–30% incidence and may result in pain, reduced quality of life and high healthcare costs [2–4]. Several suture materials and techniques for the closure of the *linea alba* after midline incisions have been investigated, however, there is still a need for closure techniques that can prevent incisional hernia [5]. The current recommendation, also stemming from a recent randomized controlled trial, is to use the small bites technique (i.e., 5 millimetre (mm) tissue bites and 5 mm between two sutures) with slowly absorbable suture materials for the closure of the *linea alba* after midline laparotomy [6]. Nevertheless, this randomized controlled trial showed that the occurrence of an incisional hernia still persists in 13% after a 1-year follow-up [6]. This result confirms that the exact biomechanical basis underlying the superiority of the small bites technique remains unknown.

In a rodent model, the dynamic change of the surgical suture tension has been investigated with the use of a customised force sensor [7]. The development of a comparable suture force or tension sensor permitted researchers and surgeons to gather data on suture tension in various tissues, suture materials, suturing patterns and closure techniques. A suture may pull through tissue due to localized pressure or tension which may cut through tissue immediately resulting in a burst abdomen or an incisional hernia after a period of time from disturbed healing by infection and/or tissue necrosis [8, 9]. Dumanian and colleagues created a novel suture of uncoated mid-weight macroporous polypropylene mesh—named Duramesh™ suturable mesh suture—to reduce the occurrence of sutures pulling through tissue and to prevent incisional hernia formation [8].

The aim of this study was therefore to measure suture tension using the small bites technique and the newly developed Duramesh™ size 0 (2 mm) also in comparison with the large bites technique, by using an implantable suture tension sensor which was developed specifically for these experiments. Furthermore, the small and large bites techniques were compared with a conventional suture material, i.e., PDS II 2-0, as a control suture material. All experiments were performed in an ex vivo porcine abdominal wall using the artificial ‘AbdoMan’.

## Methods

### Suture tension sensor

**Fig. 1:**
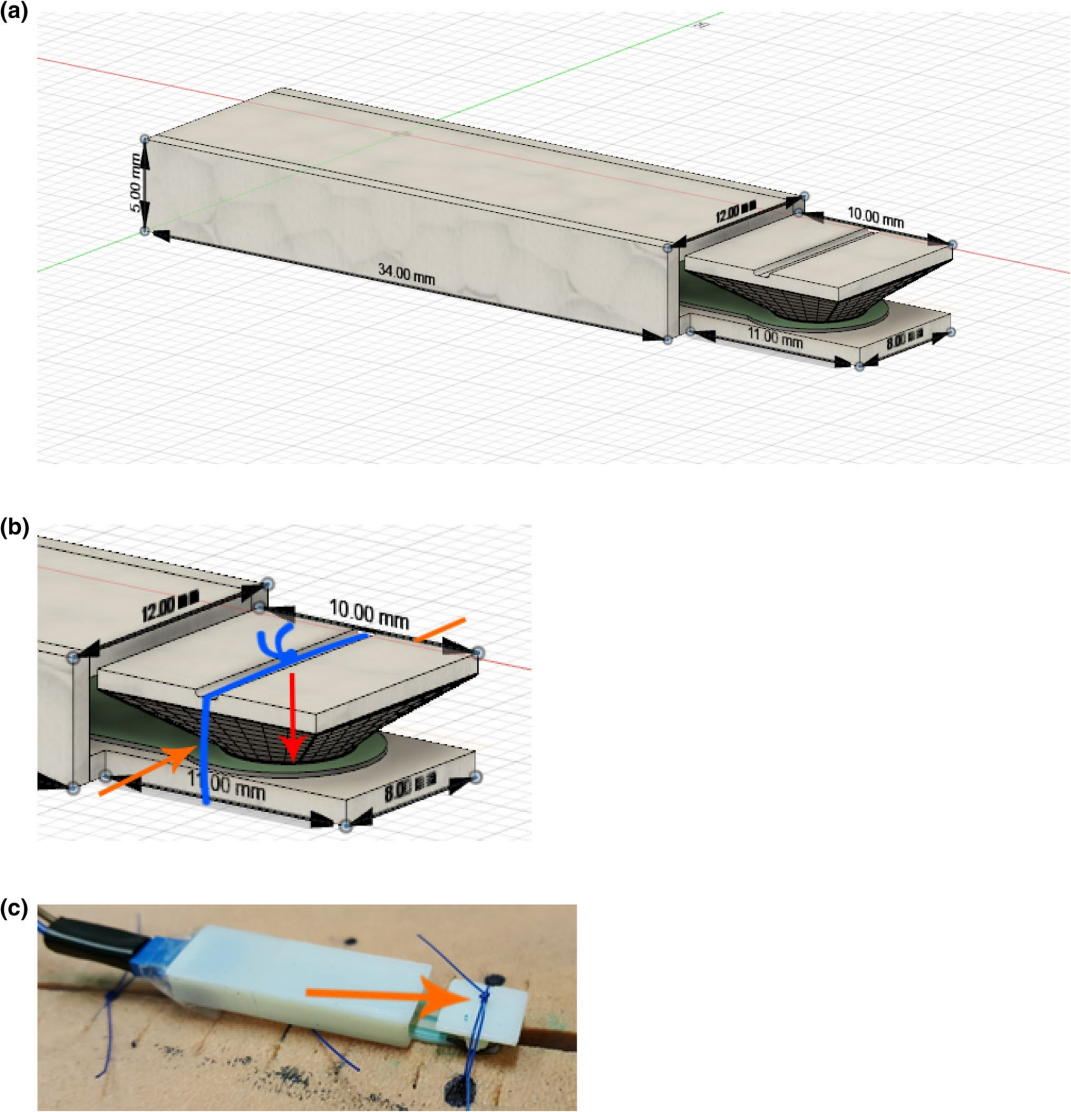
**a** Complete sensor enclosure and Interlink Electronics FSR 400 (in green). Total probe dimensions: 45 mm × 12 mm × 5 mm. **b** The tension in the suture (orange arrows) is translated to a downward force, applied to the suture tension sensor (red arrow). **c** The suture tension sensor in an experimental set-up with an artificial abdominal wall and PDS-II 2-0 single suture. The tension in the suture marked by the orange arrow was measured

An implantable suture tension sensor was developed using a Force Sensing Resistor (Interlink Electronics FSR 400, Interlink Electronics, Westlake Village, CA, USA) with an actuation force of approximately 0.2–20 N [10]. A three-dimensional (3D) model was developed for the enclosure of the suture tension sensor (Fig. 1a). The tension generated by the suture in the actuator notch is translated downward onto a circular surface, precisely and evenly pressing down on a force sensor within the suture tension sensor (Fig. 1b, c). An analog-to-digital converter (ADC) and an Arduino Uno controller (Arduino AG, Somerville, MA, USA) were used to read the raw output from the suture tension sensor. A custom-made program was written to create a live graph of the tension sensor data in Newtons (N).

### Measuring model

The ‘AbdoMan’ was developed as an artificial simulation of the human abdominal wall by taking the muscle contractions and intra-abdominal pressure into account [11]. In these current experiments, only the intra-abdominal pressure was considered. A 3500 ml air-filled collecting bag was placed on a three-dimensional printed part in the shape of an abdominal wall. A laparoscopic insufflator (Olympus UHI-3 High Flow Insufflator, Olympus Corporation, Shinjuku, Japan) was used to apply insufflation pressures up to 20 millimetres of mercury (mmHg). After sensor placement and prior to insufflation, a baseline suture tension was measured over the course of one minute. Validation of the suture tension sensor was performed before these experiments, by varying the force applied to the suture tension sensor in a controlled manner, verifying whether the suture tension sensor would be able to correctly detect and measure these variations. The measured suture tension is relative to the baseline tension in the closed incision. The measurements are a derivative of the actual tension within the suture.

### Duramesh™

Duramesh™ is a novel suturing concept, based on the principles of meshes, used in hernia repair, while providing the precision and flexibility of a suture [12]. It is a non-resorbable suture, made of polypropylene. The three-dimensional macroporous structure has a larger surface than standard sutures and it has been shown to stimulate better tissue integration in an in vivo porcine model [12].

### Experimental set-up

**Fig. 2:**
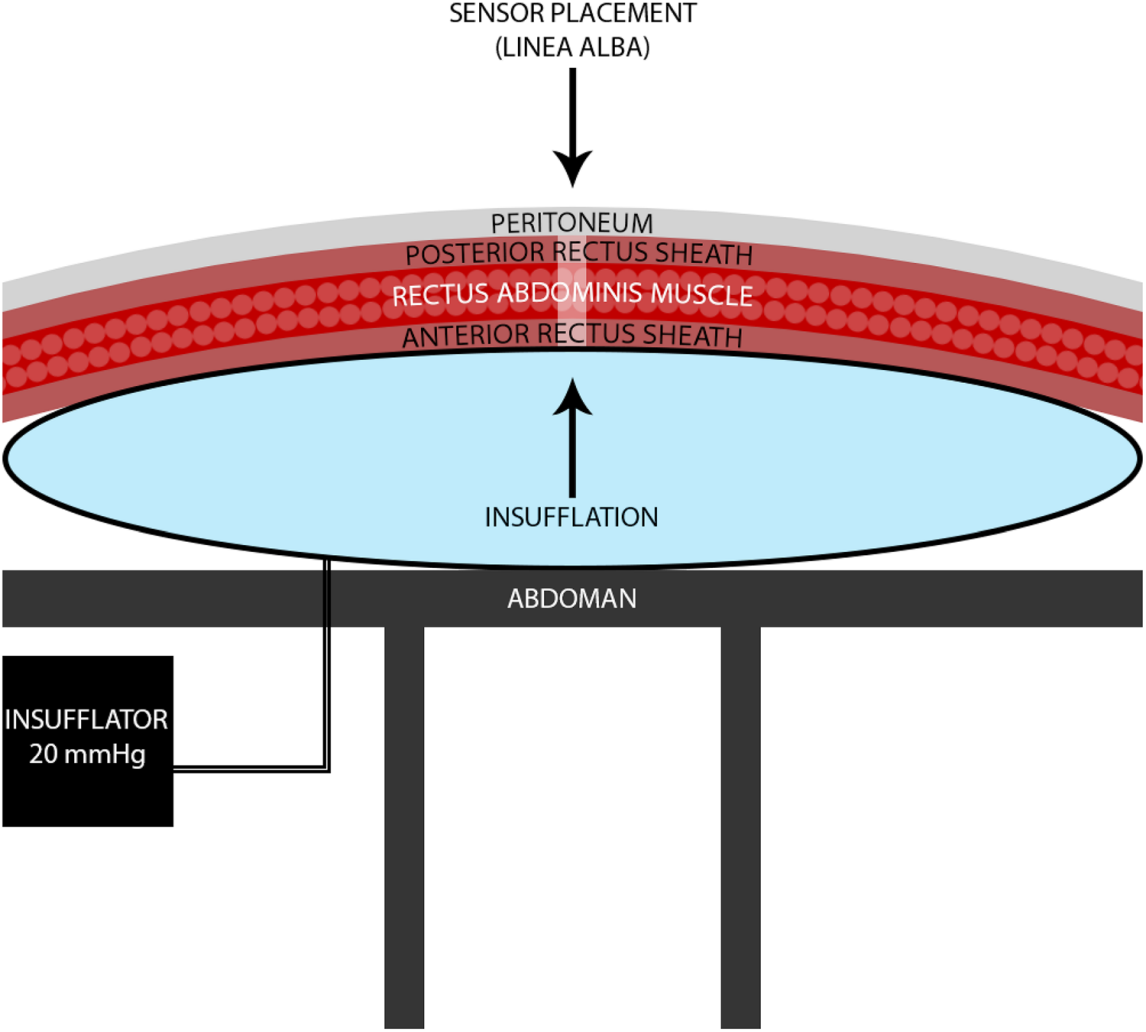
Experimental set-up

Six porcine abdominal walls of female Yorkshire-Landrace pigs with comparable dimensions, ranging in weight from 30 to 40 kilograms (kg) were explanted directly after euthanasia and frozen at −20 Celcius (°C). Twenty-four hours prior to the experiments, the abdominal wall was thawed [13]. Two midline incisions of 5 cm each (i.e., cranial and caudal) were made through all layers of the abdominal wall. This number of midline incisions and their length was chosen, because this length would be the longest possible length compatible with all specimens. The abdominal wall was inversely placed with the peritoneum upwards and fixated onto the’AbdoMan’ (Fig. 2). The *linea alba* was closed with continuous sutures including all layers of the abdominal wall, including the peritoneum. Closure was performed using the small bites (i.e., 5 mm between two consecutive stitches and 5 mm distance from the incision) and the large bites (i.e., 10 mm between two consecutive stitches and 10 mm distance from the incision) techniques with Duramesh™ size 0, 2 mm (Duramesh™ Suturable Mesh, Inc., Dorado, Puerto Rico, USA) and PDS II 2-0 (Ethicon, Somerville, NJ, USA). Locations (i.e., cranial and caudal) were switched for small and large bites to randomize for location. Each experiment was repeated twice, using the existing incisions. The suture tension sensor was placed in the middle of the incision. All experiments were performed by a single researcher. Lastly, the collecting bag was insufflated to 20 mmHg for a duration of 30 minutes (min).

Finally, the suture tension was compared between:

1. Small bites with Duramesh™ size 0 versu*s *large bites with Duramesh™ size 0 (*N* = 12).
2. Small bites with PDS II 2-0 versus large bites with PDS II 2-0 (*N* = 12).
3. Small bites versus large bites with both materials.
4. Small bites with PDS II 2-0 versus small bites with Duramesh™ size 0.
5. Large bites with PDS II 2-0 versus large bites with Duramesh™ size 0.

### Data analyses

Results are presented as median differences and interquartile ranges in suture tension. Statistical significance was assessed using a Wilcoxon rank-sum test for all samples comparing two different modalities (i.e., small bites, large bites, Duramesh™ size 0, and PDS II 2-0) after 25 min. *p* values lower than 0.05 were considered statistically significant. Python for Windows, version 3.5.1. (Python Software Foundation, Beaverton, USA) was used to perform all statistical analyses.

## Results

**Fig. 3:**
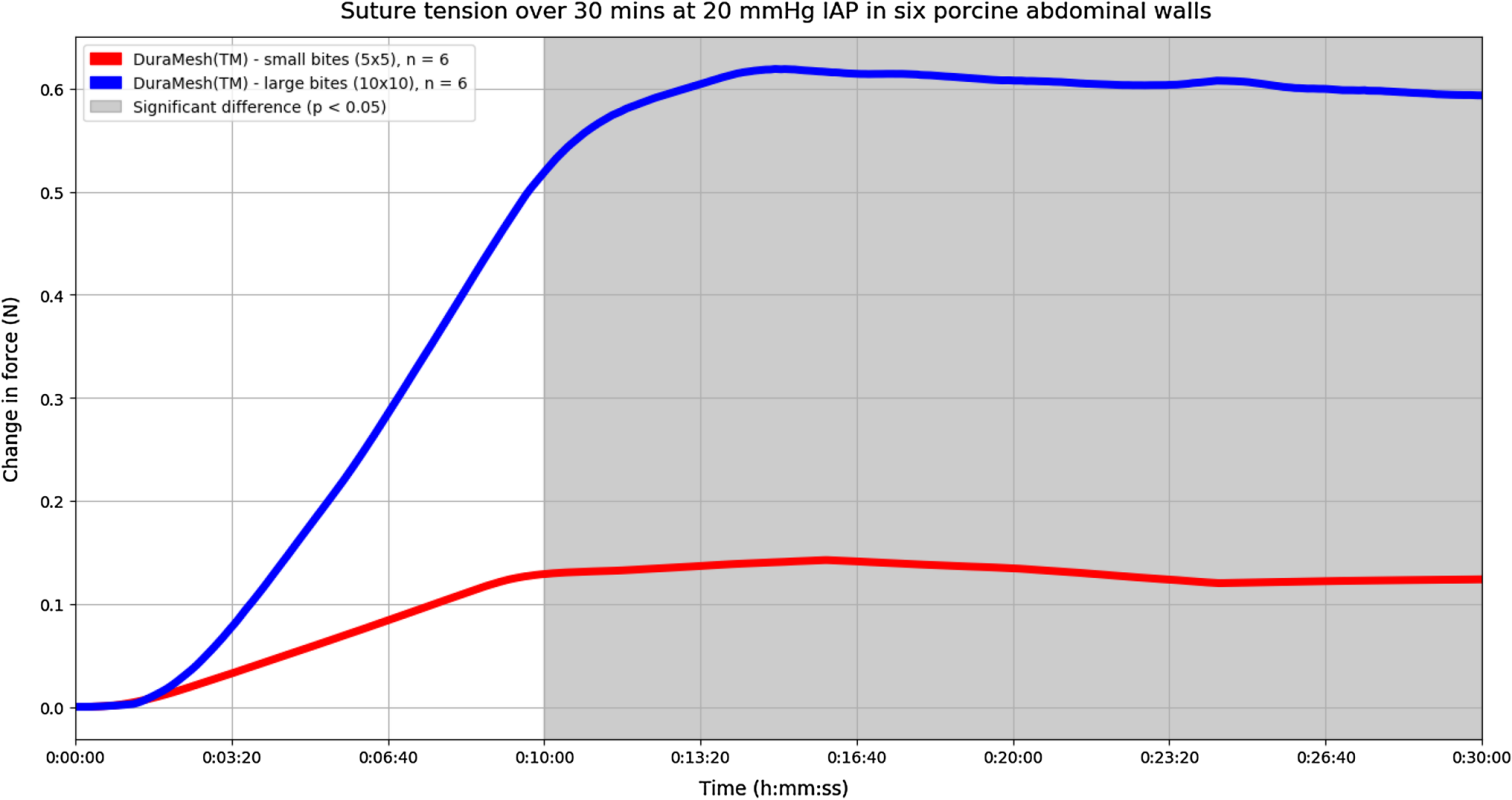
Experiments performed with only Duramesh™ size 0. The median change in suture tension of all small bites is shown by the red line. The median change in suture tension of all large bites is shown by the blue line. Small bites were significantly more efficient in dividing suture tension across the incision when compared to large bites at time points in the shaded area

**Fig. 4:**
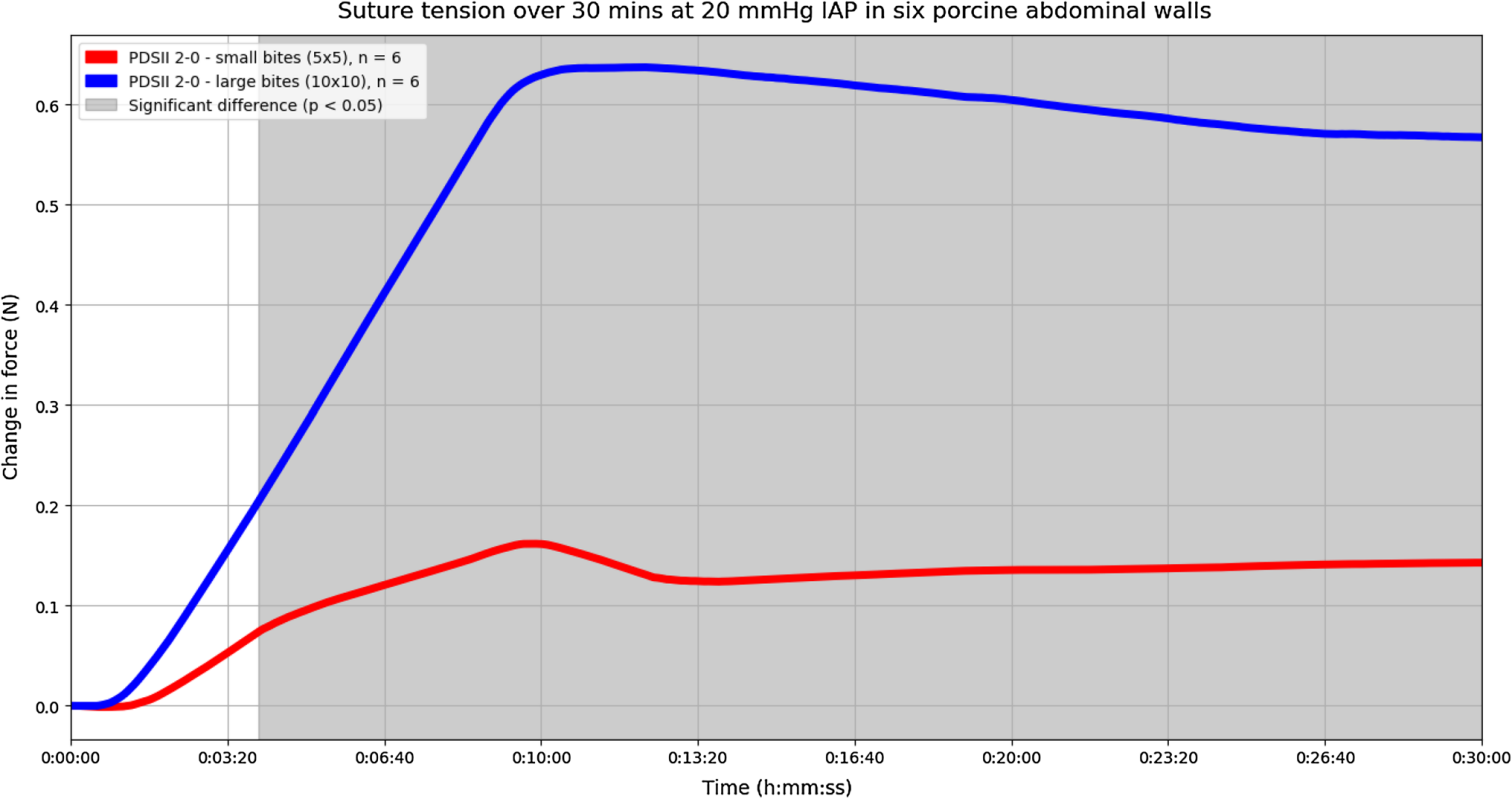
Experiments performed with only PDS II 2-0. The mean change in suture tension of all small bites in red. The mean change in suture tension of all large bites in blue. Small bites were significantly more efficient in dividing suture tension across the incision when compared to large bites, at time points in the shaded area

In total, 24 experiments were performed. No macroscopic tissue failure was visible during or after the experiments. Median suture tension was calculated for one point in time; i.e., at 25 min from the start of insufflation, when the suture tension had reached a plateau in all experiments. When considering the two suture materials individually, each showed a significant difference in suture tension between small and large bites, in favour of the small bites. Regarding Duramesh™ size 0: small bites 0.12 N (IQR 0.07–0.19) versus large bites 0.57 N (IQR 0.23–0.92), *p*  < 0.025 (Fig. 3). Regarding PDS II 2-0: small bites 0.15 N (IQR 0.05–0.31) versus large bites 0.56 N (IQR 0.37–0.98), *p* < 0.038 (Fig. 4).

**Fig. 5:**
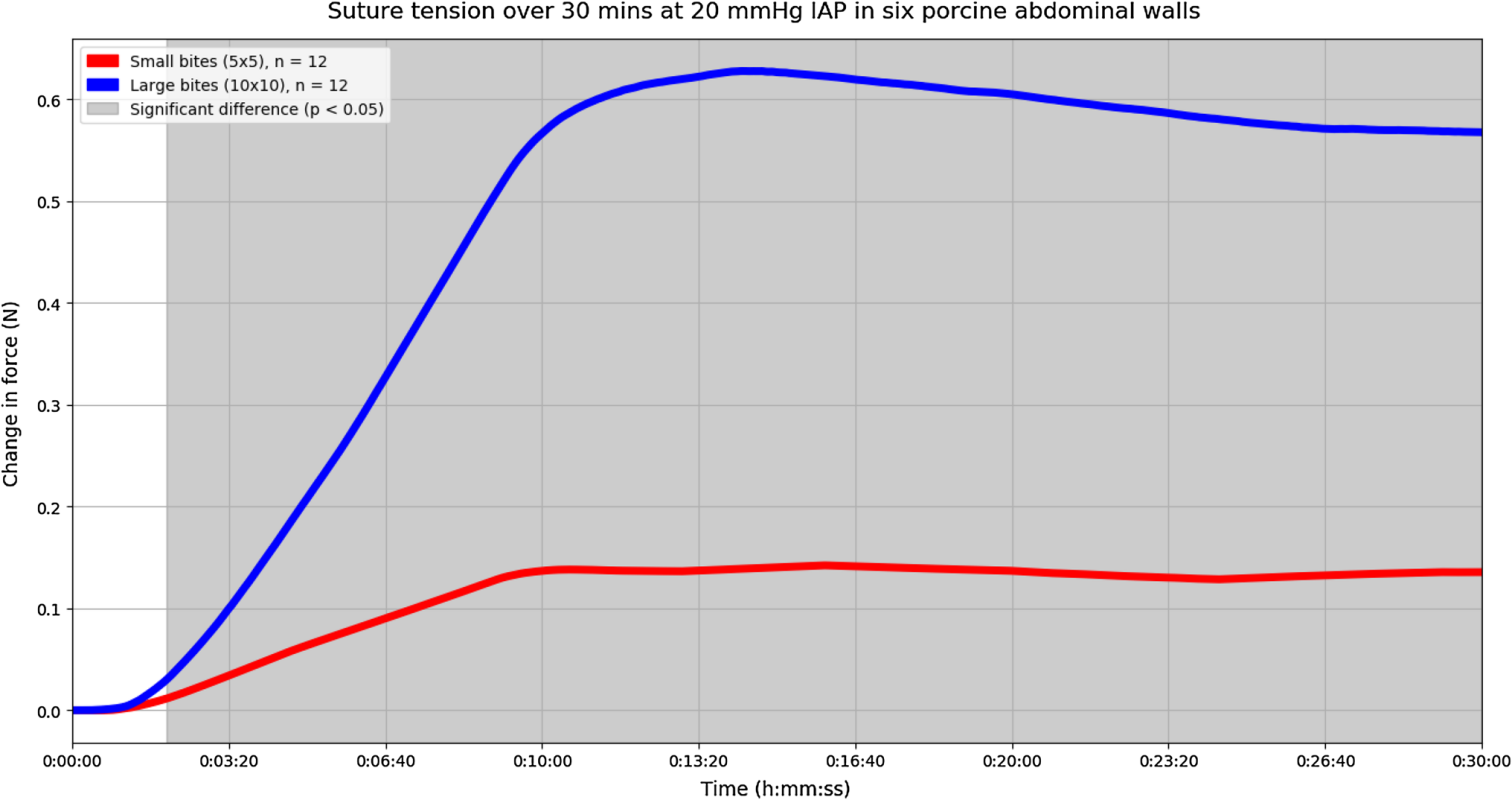
Experiments performed with both suture materials. The median change in suture tension of all small bites is shown in red. The median change in suture tension of all large bites is shown in blue. Small bites were significantly more efficient in dividing suture tension across the incision when compared to large bites, at time points in the shaded area

Irrespective of the suture material used, the tension in the sutures was significantly lower when the *linea alba* had been closed with small bites when compared to the tension in the sutures when large bites had been applied (small bites 0.14 N (IQR 0.06–0.20) versus large bites 0.56 N (IQR 0.31–0.98), *p* < 0.0015, Fig. 5).

When only considering small bites, there was no significant difference in suture tension between Duramesh™ size 0 and PDS II 2-0 (Duramesh™ size 0 0.12 N (IQR 0.07–0.19) versus PDS II 2-0 0.15 N (IQR 0.05–0.31), *p* > 0.05, Supplemental Fig. 1). Similarly, when only considering large bites, there was no significant difference in suture tension between Duramesh™ size 0 and PDS II 2-0 (Duramesh™ size 0 0.57 N (IQR 0.23–0.92) versus PDS II 2-0 0.56 N (IQR 0.37–0.98), *p* > 0.05, Supplemental Fig. 2).

## Discussion

In this present study, small bites and the use of Duramesh™ resulted in a significantly lower suture tension when compared to the tension in the sutures in large bites in a porcine abdominal wall. The suture tension was measured with a custom-made suture tension sensor in an experimental set-up using the artificial ‘AbdoMan’. This significant difference was also found when the same comparison was made with the use of PDS II 2-0 as a control suture material. These findings were analogous to findings from a recent clinical study [6]. The superiority of the small bites technique is not limited to PDS II 2-0 sutures, but also holds for suture materials with an elaborate three-dimensional structure, such as the Duramesh™ size 0 and perhaps for other types of suture materials. However, in this present experiment Duramesh™ size 0 was neither superior, nor inferior, compared to PDS II 2-0. This finding makes Duramesh™ a viable option in choosing suture materials for abdominal wall closure. Nevertheless, most previous experiments with Duramesh™ have revolved around linearly pulling it until either tissue or suture failure [12, 14]. In this experimental set-up, Duramesh™ was tested to much weaker forces i.e., 20 mmHg. Simulating pulling to failure in this set-up would involve raising the intra-abdominal pressure to (much) higher levels than 20 mmHg, perhaps ranging in the hundreds of mmHg. Therefore, a tensile test would be more suitable for this kind of experiments to demonstrate a difference. When being pulled, the Duramesh™ size 0 stretched and flattened like a ribbon, which may be helpful in dividing the suture tension across the wound. When the Duramesh™ size 0 was pulled through the tissue, the structure of the suture was occasionally damaged. This damage may have compromised the integrity of its shape, thereby impairing its mechanism of action. That being said, the Duramesh™ size 0 or PDS II 2-0 never broke completely. It should be noted that only size 0 (2 mm) of the Duramesh™ was tested in these present experiments. The expectation is that the superiority of Duramesh™ will be clearer in an in vivo model, where tissue integration can be measured as well.

The relation between suture material and the development of burst abdomen has not been studied extensively. Van Ramshorst et al. found that failure of the knot was a significant cause in addition to other causes like ileus [1]. On the contrary, the effects of different suture materials, suture configurations and suture length to wound length ratio on the occurrence of incisional hernia have been extensively studied [5, 15]. Cooney et al. found the best performing bite separation and bite width to be 5 and 16 mm, respectively, in a biomechanical abdominal wall model [16]. As they also state, this is partly in agreement with the findings of the STITCH trial. However, they suggest that perhaps a small bite separation should be combined with a large bite depth rather than a small bite depth like the 5 mm used in the STITCH trial. Comparing these two modalities should be the next step in this present experimental setup. However, the optimal suture tension has not been studied largely [7]. The tension in a suture is the composite result of the type or resistance of tissue, the suture material used, the force applied by the surgeon during knotting, and the bite width and separation [7]. Proper closure of the abdominal wall involves the close approximation of tissue edges with sutures. If the sutures are too loose, however, the wound edges cannot be properly approximated and there will exist a risk for impaired healing and wound dehiscence. This theoretically would result in an increased risk for incisional hernia formation [1]. Another reason for insufficient suture tension could be the phenomenon of creep, in which the suture will be irreversibly elongated over time as a result of a continuous pulling force [10]. Inadequate abdominal fascial closure may also be seen in cases where the tension in the sutures is too high. The suture may cut through tissue and cause additional tissue damage, tissue necrosis or an incisional hernia [1]. This implies that the relation between suture tension and outcome is parabolic, allowing for the definition of a possibly optimal suture tension [17]. Nonetheless, it is not easy to obtain the ideal suture tension since this is subject to inter- and intra-surgeon variability [18]. It would be helpful to have a device attached to the suture needle or the suture material continuously measuring suture tension so that the surgeon would be able to apply the same suture tension with every knot or throw. While there is no currently available method to determine suture tension during suturing, the pore size of the Duramesh™ was macroscopically changed with higher tension, giving the surgeon feedback while suturing.

As almost in every in vitro study, this study also has limitations. One limitation in this study is the use of porcine abdominal walls instead of human abdominal walls, which were not available. However, in a recently published study, porcine tissue was demonstrated to be an appropriate surrogate for examining the human abdominal wall when it comes to the *linea alba* [19]. Another limitation was that the suture length to wound length ratio of at least 4:1 was established prior to the experiment. However, with the small bites technique, twice as many suture loops were placed than with the large bites technique. The present ex vivo experiments can be considered an acute postoperative model rather than a wound healing model. As a consequence it can be concluded that the new Duramesh™ size 0 suture seems to behave similar to a conventional suture like PDS II 2-0, that the advantages of small bite closure of the *linea alba* also apply to it, but that it cannot be expected to prevent the early development of burst abdomen and incisional hernia in a better way. One could propose that the three-dimensional, macroporous structure of the Duramesh™ would provide for a more profound tissue integration, allowing the tissue to grow through its individual threads and completely envelop the suture. This could hypothetically strengthen the wound healing and help prevent incisional hernia, something which has already been shown in in vivo experiments [8]. Finding a way to simulate wound healing, such as in animal models, would allow to focus on incisional hernia formation at a later point in time—after closure of the abdominal wall and during the healing process. In such an experimental setup the Duramesh™ would be expected to be more efficient when compared with conventional suture materials. In the future, this experimental setup and the suture tension sensor might be used for experiments with other suture materials and configurations.

## Conclusion

The suture tension with the small bites technique and the use of Duramesh™ size 0 was significantly lower when compared with large bites in this model. Additionally, macroscopic tissue failure was not seen in either suture material during or after the experiment. Further research should be conducted to find out whether these findings are also valid in different stages of wound healing and in abdominal walls of different origins, shapes and sizes, as well as with the use of other types of sutures or suturing techniques.

## Electronic supplementary material

Below is the link to the electronic supplementary material.

Supplementary file1 (DOCX 165 kb)
