# Supplementary material for: Evaluation of a new suture material (Duramesh™) by measuring suture tension in small and large bites techniques for laparotomy closure in a porcine model
Source: Hernia. 2020 Feb 21;24(6):1317–24. doi: 10.1007/s10029-020-02140-7 (PMC7701069; doi:10.1007/s10029-020-02140-7)
Supplement: Supplementary file 1 — Supplementary file1 (DOCX 165 kb) [file 10029_2020_2140_MOESM1_ESM.docx]

**Evaluation of a new suture material (Duramesh™) by measuring suture tension in small and large bites techniques for laparotomy closure in a porcine model**

Y. Yurtkap, MD^1*^, F.P.J. den Hartog, BSc^1*^, W. van Weteringen, MD^2^, J.J. Jeekel, MD, PhD^3^, G.J. Kleinrensink, PhD^3^, J.F. Lange, MD, PhD^1^

* Both authors contributed equally and both authors should be considered as first author.

1. Erasmus University Medical Centre, Department of Surgery, Rotterdam, the Netherlands
2. Erasmus University Medical Centre, Sophia Children’s Hospital, Department of Paediatric Surgery, Rotterdam, the Netherlands
3. Erasmus University Medical Centre, Department of Neuroscience and Anatomy, Rotterdam, the Netherlands

**Corresponding author**Y. Yurtkap
Erasmus MC, Dr. Molewaterplein 40, 3015 GD, Rotterdam, The Netherlands
[y.yurtkap@erasmusmc.nl](mailto:y.yurtkap@erasmusmc.nl)

**Supplemental materials**

**
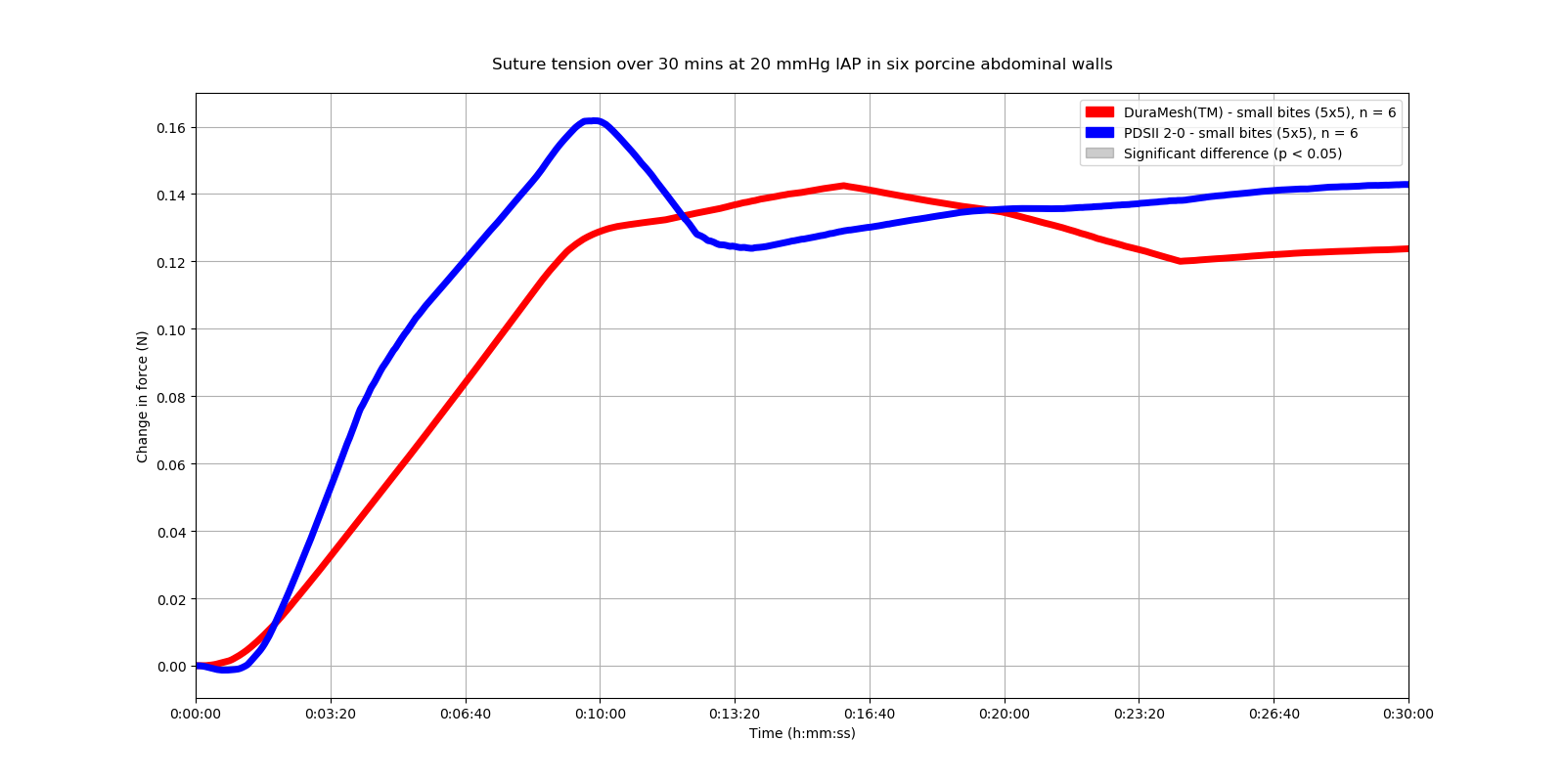
**

**Figure 1:** No significant difference in suture tension between Duramesh™ size 0 and PDS II 2-0 was seen when only considering small bites.

**
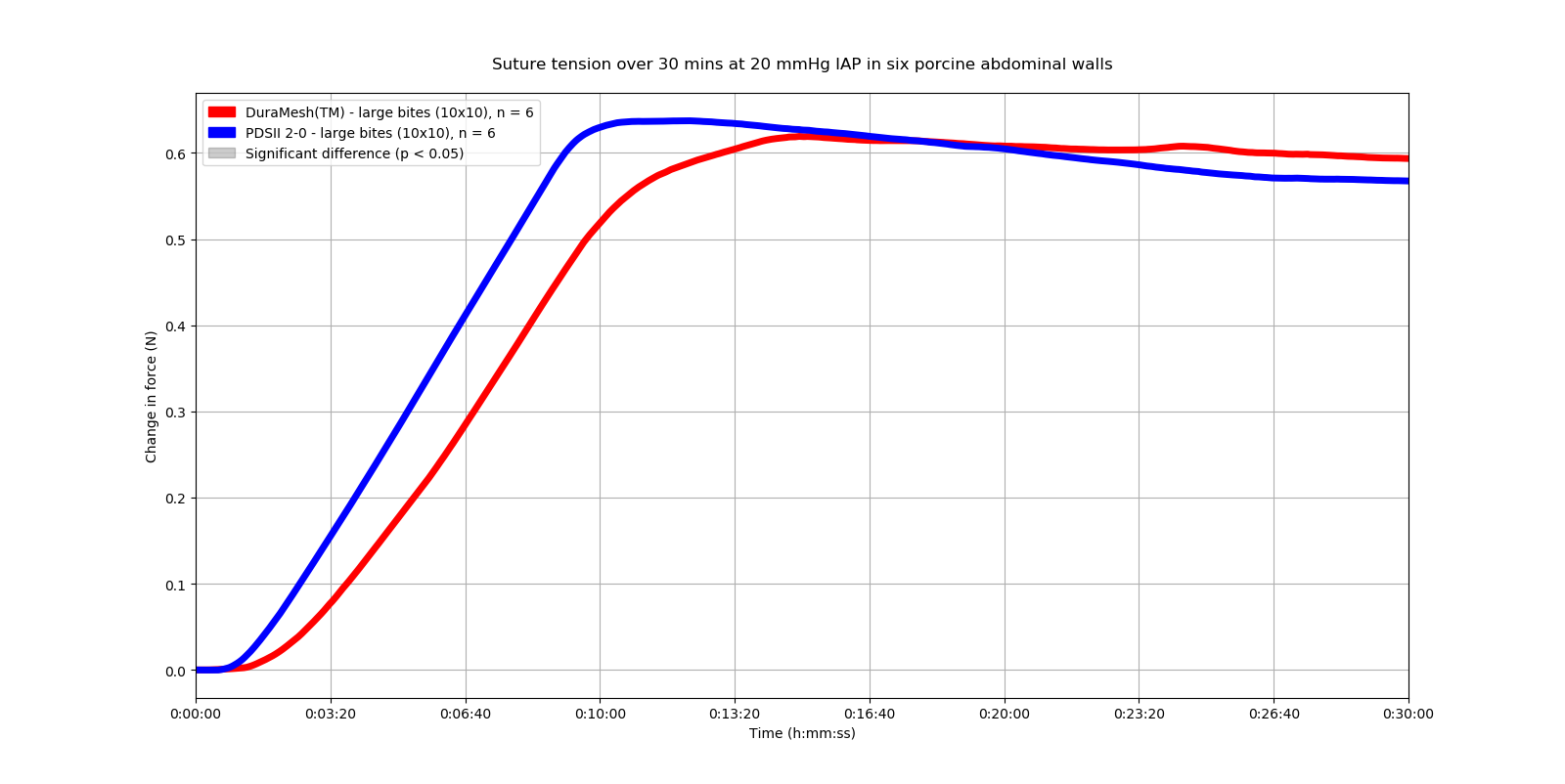
**

**Figure 2:** No significant difference in suture tension was seen between Duramesh™ size 0 and PDS II 2-0 when only considering large bites.
